# Value of Adding Bioelectrical Impedance Analysis to Anthropometric Indices in the Diagnosis of Metabolic Syndrome in 10–16 Years Old Schoolgirls

**DOI:** 10.3390/healthcare10030419

**Published:** 2022-02-23

**Authors:** Rawan G. Muhanna, Ghadeer S. Aljuraiban, Najwa K. Almadani, Mohammed Alquraishi, Mohamed S. El-Sharkawy, Mahmoud M. A. Abulmeaty

**Affiliations:** 1 Department of Community Health Sciences, College of Applied Medical Sciences, King Saud University, Riyadh P.O. Box 11362, Saudi Arabia; rawan.muh86@gmail.com (R.G.M.); galjuraiban@ksu.edu.sa (G.S.A.); anajwa@ksu.edu.sa (N.K.A.); malquraishii@ksu.edu.sa (M.A.); 2 Department of Radiology and Medical Imaging, College of Medicine, King Khalid University Hospital, King Saud University, Riyadh P.O. Box 11922, Saudi Arabia; msherif@ksu.edu.sa

**Keywords:** metabolic syndrome, anthropometry, BIA, schoolgirls

## Abstract

The use of bioelectrical impedance analysis (BIA) in clinical settings is common. However, the value of BIA-based parameters in diagnosing metabolic syndrome (MetS) in children is under-investigated. Herein, we aimed to study the usefulness of BIA-indices in the diagnoses of MetS in 6–10-year-old girls. Therefore, a diagnostic accuracy case-control study was conducted, which included 75 girls aged 10–16 years, divided into three age-matched groups (normal, None-MetS, and MetS). Anthropometric indices, BIA parameters (including fat-free mass (FFM), body fat percent (BFP), and total body water (TBW)), blood pressure (BP), and blood samples were collected. Our main findings show that for girls in None-MetS and MetS groups, the waist circumference (WC) correlated positively with waist-hip ratio and mid-arm circumference (r = 0.58, 0.47, respectively), but not with BFP based on skinfold thickness (SFT), or mid-arm muscle area. WC was positively correlated with FFM and TBW, while high-density lipoprotein was inversely correlated with FFM. However, fasting blood glucose, triglycerides and BP showed no association with anthropometric measurements and BIA components. WC was the best indicator of MetS (AUC = 0.88, cut-off = 81.5 cm), followed by BMI (AUC = 0.84, cut-off = 26.9 kg/m^2^), while BFP based on SFT was the least sensitive (62.5%). In conclusion, apart from the FM index, anthropometric parameters such as WC are more valuable in diagnosing MetS in young adolescent girls.

## 1. Introduction

It is well established that uncontrolled adiposity is a major risk factor for coronary heart diseases, diabetes mellitus, mental illnesses, and some types of cancer [1]. Metabolic abnormalities such as dyslipidemia, high blood pressure (BP), and high fasting blood glucose (FBG) are often provoked by excess weight and obesity [2]. The clustering of these metabolic abnormalities, along with central obesity, has been termed “metabolic syndrome” (MetS) [3,4]. Though MetS is not present in all individuals with obesity, it is increasing at an alarming rate in adults and children, especially in the Middle East, in parallel with the obesity epidemic [3,4], leading to doubled cardiovascular disease (CVD) risk [5]. A systematic review revealed that the median prevalence of MetS was 3% in children, 12% in children that were overweight, and 29% in obese children [6].

Since elevated visceral adipose tissue is the hallmark of MetS and is highly reversible, accurate diagnosis of visceral adipose tissue can be an effective preventive approach [7]. Currently, the use of conventional anthropometric measurements alone, such as waist circumference (WC) and age-and-gender percentile body mass index (BMI) for predicting MetS are controversial, given that BMI percentiles are not based on an increased risk of cardiometabolic endpoints [8,9]. Further, BMI does not reflect actual body composition or indicate abdominal obesity [5]. Moreover, the International Diabetes Federation (IDF) considers abdominal obesity (≥90th percentile or adult cut-off if lower) as a cornerstone of the factors required to diagnose MetS in children and adolescents 10 to <16 years of age [10]. However, the American Heart Association and the National Heart, Lung and Blood Institute do not consider abdominal obesity as a mandatory risk factor for MetS and use a different cut-off of WC ≥102 cm for males and ≥88 cm for females (in adults and children), using data obtained from Western sample populations [11] which may not be generalizable to all regions around the world. Further, other components of MetS (FBG, triglycerides (TG), and high-density lipoprotein (HDL)) require relatively invasive and expensive tests and may not be accessible in all settings. In search of reliable and less invasive methods to predict MetS, Bioelectrical Impedance Analysis (BIA), a validated tool that measures fat mass (FM), fat-free mass (FFM), central obesity, and other body components, was introduced in epidemiological studies [12,13,14]. However, its use in diagnosing MetS and obesity in children and adolescents, especially in the Middle East, has not been thoroughly investigated. It is also unclear whether measurements of general or central obesity are suitable for identifying MetS in children and adolescents. Thus, there is a need to identify which anthropometric parameter provides the best predictive value for MetS.

The study’s primary objective was to compare anthropometric and BIA indices for diagnosing MetS in children and adolescents living in Riyadh, Saudi Arabia. We also aimed to compare the sensitivity and specificity of different indices and provide cut-off values for predicting MetS in a sample of female school children and adolescents aged 10–16 years.

## 2. Materials and Methods

### 2.1. Study Design

The present diagnostic accuracy case-control study was conducted in three randomly selected schools from a list of girls’ schools in Riyadh city starting from December 2017 for six months. These schools were located in three different zones of Riyadh city and included a governmental school, a national, and an international school. After obtaining approvals from the school administration, eligible female students aged 10–16 y were invited to participate in this study by sending letters to parents explaining the purpose and process of participation. After approvals were granted from the parents, students were screened for eligibility. Participants were divided into three age-matched groups (Normal, None-MetS, and MetS). The normal group included normal-weight girls (5th percentile to less than 85th percentile) [15] without any apparent diseases; the None-MetS group included girls with overweight/obesity and without MetS (girls with BMI above the 95th percentile for age, without other criteria of MetS according to IDF) [15], and in the MetS group girls with Obesity and MetS were included (meeting the IDF criteria of MetS; WC ≥ 90th percentile or adult cut-off if lower, triglycerides (TG) ≥ 1.7 mmol/L, HDL-C < 1.03 mmol/L, systolic ≥ 130 and diastolic ≥ 85 mm Hg, FBG ≥ 5.6 mmol/L) [10].

**Figure 1:**
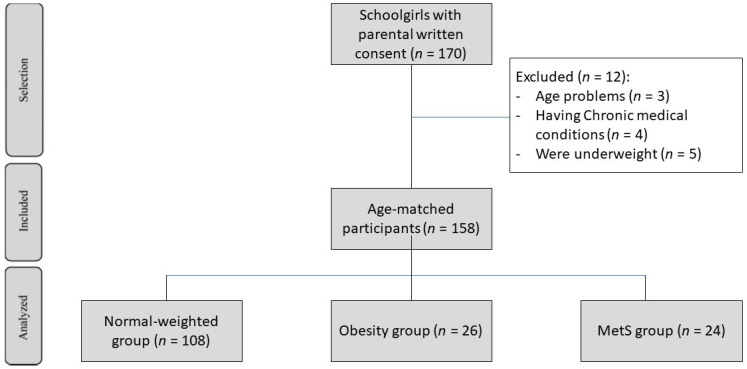
Flow chart of the study progress.

Potential participants were excluded (*n* = 12) if they had any chronic medical conditions (other than obesity or MetS) such as type 1 diabetes, were underweight (<5th percentile), were below the age of ten, or over the age of sixteen, in order to align with the IDF criteria. In addition, potential participants who received medication that might affect the accuracy of the measurements, such as those altering BP, glucose, or lipid metabolism, as well as medication causing water retention (Figure 1), were also excluded. Ethical approval was obtained by the local Ethics Committee of the College of Applied Medical Sciences, Department of Community Health Sciences, King Saud University in Riyadh, Saudi Arabia, under reference number CMS 155-36/37.

Eligible participants attended the school clinic with one parent after an overnight fast (minimum of 8 h). During the visit, a trained person administered a health history questionnaire to collect demographic and socioeconomic information (age, the order in the family, number of siblings, known allergy or health problem, physical activity, screen time, sleep duration, activity preferences, parents’ educational level, occupation, monthly income, history of chronic diseases or obesity), obtained anthropometric measurements, collected BP readings, withdrew blood samples for biochemical tests, and conducted BIA. Additional details on the data collection are provided below.

### 2.2. Anthropometric Measurements

Bodyweight and height were measured to the nearest 0.1 in kg and 0.1 cm, respectively, without shoes and light clothes. WC was measured at the midpoint between the lowest rib margin and the iliac crest and recorded to the nearest 0.1 cm. Hip circumference (HC) was measured around the widest portion of the buttocks, recorded to the nearest 0.1 cm. An average of two consequent measurements was used for analysis. For purposes of defining criteria and new cut-offs, we also included other measurements such as mid-arm circumference (MAC), where the measuring tape was placed at the midpoint between the upper edge of the posterior border of the acromion process of the scapula and the posterior surface of the arm to the tip of the olecranon process (the bony part of the mid-elbow), and other measures were recorded to the nearest 0.1 cm. Skin Fold Thickness (SFT) was measured at four different areas of the body (triceps, biceps, abdomen, and subscapular), recorded to the nearest 0.1 mm, and body fat percent based on the SFT (BFP-SFT) was calculated using the Slaughter equation [16,17]. The Mid-Arm Muscle Area (MAMA) was calculated according to Soler-Cataluña (2005) as follows (MAC (cm)) − 3.14 × tricipital skinfold thickness (mm)) 2/(4 × 3.14). All measures were collected twice to ensure accuracy, with an average of two measures used in the analysis.

### 2.3. Blood Pressure and Biochemical Measures

BP was measured from the right arm using a sphygmomanometer with an inflatable cuff while the participant was sitting (OMRON M6). FBG level was measured using the common glucometer, ACCU-CHEK Active. A sample of 15–40 µL of blood was collected from each participant by a trained nurse to measure fasting TG levels, HDL, and total cholesterol by a small portable device (CardioChek PTS Inc., Whitestown, IN, USA).

### 2.4. Bioelectrical Impedance Analysis

Participants were instructed to clean the soles of their feet and palms with electrolyte tissue, then stand barefoot on the lower limb electrodes and hold the upper limb electrodes of the BIA device (Tanita BC-418, Tanita Corporation, Tokyo, Japan) to provide detailed body composition data through the use of 8 polar electrodes in less than 30 s. Recorded parameters included TBW, body fat percent (BFP), FM, and FFM (FM and FFM were calculated in kilograms divided by the square of the height in meters, respectively) [18].

### 2.5. Statistical Analysis

The sample size was calculated based on the hypothetical proportion of normal-weighted group exposed to having MetS of about 0.2%, and the hypothetical proportion of None-MetS cases with exposure of about 27% [19,20], with a statistical power (1-beta, % chance of detecting) of 80% and two-sided significance level (1-alpha) of 95%. The required estimated sample size for comparing the IDF and BIA for identifying MetS was 170 participants.

Quantitative data were summarized using the mean, standard deviation (SD), and range, while categorical variables were summarized as percentages, presented per normal, obese, and MetS participants. One-way analysis of variance (ANOVA) with a post-hoc test (Fisher’s Least Significant Difference) was used to compare the means of the three study groups. A chi-squared test was used to compare percentages of categorical variables. Pearson’s correlation coefficient was used to identify the correlation between the study parameters. Receiver operating characteristics curves and areas under the curve (AUCs) were used to detect obesity and identify new cut-points with the highest sensitivity (true positive rate) and specificity (true negative rate) of BMI, WC, HC, BFP-SFT, MAMA, BFP, FMI, FFM, and TBW. At *p* ≤ 0.05, the value was considered to be statistically significant. Statistical analyses were performed using SPSS for Windows (version 23; IBM, Armonk, NY, USA).

## 3. Results

### 3.1. Participant Characteristics

Out of 170 screened participants, 158 girls completed all study parameters; normal group (*n* = 108), None-MetS (*n* = 26), and MetS (*n* = 24). All participants were between the ages of 10 and 16, with a mean age of 13.3 ± 2.6 years.

Both parents in all groups had received a high level of education (university or higher), with a slightly higher percentage in the MetS group (25.3% for the mother and 32% for the father). Current findings show, that in comparison to girls with normal BMI, BFP was significantly higher in both None-MetS and MetS groups in comparison to the normal-weight group (*p* < 0.01); however, the difference between None-MetS and MetS was not significant (*p* = 0.12). Compared to girls with normal BMI, FBG and TG were significantly higher, while HDL was lower in the MetS group (*p* < 0.05) (Table 1).

### 3.2. Correlations of Anthropometric Measurements and BIA with the Components of MetS

For anthropometric measurements in the None-MetS group, WC was highly correlated with BMI, HC, WHR, and MAC (r = 0.47, 0.65, 0.58, 0.47, respectively) (Table 2). For BIA components, WC was highly correlated with FFM and TBW (r = 0.49, 0.57, respectively). HDL level was inversely correlated with FFM (r = −0.44). In the MetS group, WC was highly correlated with BMI (r = 0.54), HC (r = 0.67), and WHR (r = 0.45). FBG was inversely correlated with BFP (r = −0.43), while SBP was directly correlated with BFP (r = 0.50). For BIA components, WC was highly correlated with FFM (r = 0.50). Other components showed no correlation with BIA components.

### 3.3. Sensitivity and Specificity of Anthropometric Measures and BIA Components

**Figure 2:**
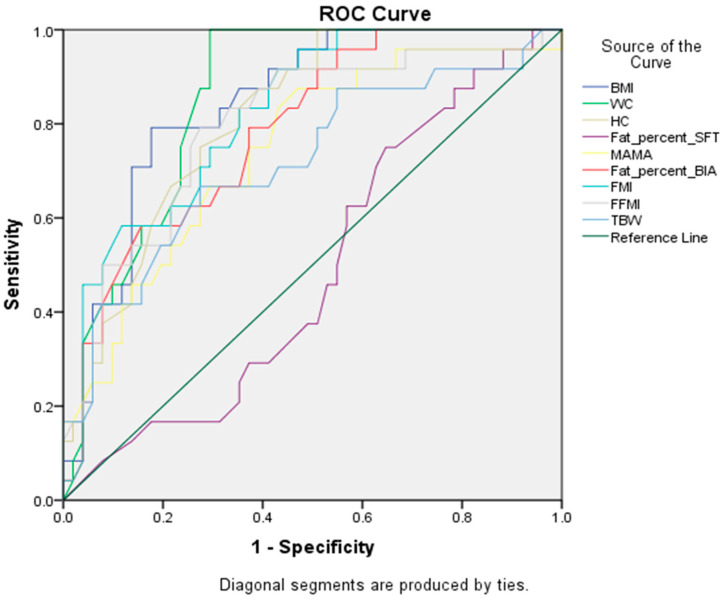
Receiver Operating Characteristics (ROC) curve showing sensitivity and specificity of BMI, WC, HC, BFP-SFT, MAMA, BFP, FMI, FFMI, TBW at different cutoff points in addition to the area under the curve (AUC).

WC was the most sensitive measure to detect cases with MetS (AUC = 0.88) (Figure 2), setting a cut-off point of 81.5 cm for diagnosing MetS with a specificity of 72.5% (Table 3. WC was followed by BMI (AUC = 0.84) (Figure 1), showing high sensitivity and a cut-off point of 26.9 kg/m^2^ for BMI. On the other hand, BFP-SFT was the least sensitive (62.5%) among anthropometric measures. FM showed the most sensitivity by MetS, while TBW showed the least (Table 3).

## 4. Discussion

In this diagnostic accuracy study of female school children and adolescents aged 10–16 years, we compared the individual components used in the definition of MetS utilizing anthropometric measures versus BIA. We showed that in females with obesity and MetS, only WC, a MetS component, correlated positively with BMI, HC, WHR, MAC, but not with body BFP-SFT or MAMA. For BIA components, WC was positively correlated with FFM and TBW, while HDL was inversely correlated with FFM. Other components of MetS (FBG, TG, SBP, DBP) showed no association with anthropometric measurements and BIA components in the None-MetS and MetS groups. We also found that WC was the best indicator of MetS with the highest AUC and a cut-off point of 81.5 cm, followed by BMI with a cut-off point of 26.9 kg/m^2^.

Few available studies have investigated the association between anthropometric indices measured by BIA and body composition with MetS in children and adolescents [21,22]. Some observational studies showed that neck circumference [23], BFP-SFT [24], and BMI [25] were the strongest anthropometric predictors of MetS in children and adolescents in different sample populations [7,26]. However, our findings in Saudi females revealed that WC, a simple, non-invasive, and low-cost method, was best associated with anthropometric parameters and BIA components compared to other parameters.

A cross-sectional study by Ramírez-Vélez et al., including Colombian college students, revealed that only BFP and FM (using BIA) were directly correlated with MetS [27]. However, our study did not support this finding, which might be due to differences in age groups (e.g., growth in children) and the inclusion of females-only data. Other studies have noted sex differences in estimating BFP using BIA [28].

Our results suggest that the highest AUC area was for WC, indicating that this is the best indicator of MetS with a cut-off point of 81.5 cm. The BMI followed the WC with a cut-off point of 26.9 kg/m^2^. These findings are in line with a previous cross-sectional study of Saudi adults aiming to identify the sensitivity of anthropometric measures in determining major diseases, including MetS, where WC had the highest AUC area for MetS and dyslipidemia (AUC = 0.84) [29]. In a cohort of 1035 Brazilian adolescents aged 12–20 years that aimed to identify the sensitivity and cut-off points of anthropometric measures related to MetS, visceral adiposity index scored the highest AUC for MetS, followed by WC and WHtR. All tested anthropometric measures (BMI, WC, WHtR, Conicity Index) had similar AUCs with WHtR presenting the best combination of sensitivity and specificity with AUC = 0.76 for girls and 0.74 for boys [30]. It was also reported that boys and girls who scored higher than the cut-off point for WHtR, i.e., 0.48, were two to three times more likely to present MetS [30].

As for the BIA, the highest AUC area was for FM with AUC = 0.82. In the present study, it is noteworthy that FM was highly correlated with both BMI and BFP by BIA. On the other hand, there was no correlation between FM and the components of MetS. Unlike our findings, a cross-sectional study by Ramírez-Vélez et al. [27] found that FM and BFP could detect MetS in college students. The sensitivity for BFP was 96.1% and 97.4% for men and women, respectively, while the sensitivity for FM was 97.6% for females and 95.8% for males [27]. However, Ramírez-Vélez et al. studied college students, and the results may differ when tested on a younger age group due to the growth period.

Our study is one of few studies investigating anthropometric and BIA indices for diagnosing MetS in child and adolescent girls. We also compared the sensitivity and specificity of different indices and provided cut-off values for the prediction of MetS in a sample of children and young adolescents living in Riyadh, Saudi Arabia. The use of anthropometric measures, BIA, and biochemical blood tests adds strength to our findings. Our results have public health and clinical implications as children and young adolescents, especially in the Middle East, are prone to major behavioral and metabolic changes, such as the adoption of a “Western” lifestyle, which have led to a rise in the prevalence of overweight and obesity [31]. The current study findings can help identify the clinical characteristics and cut-off values early in life that could be used to screen for MetS risk. From a financial point of view, the cost of a BIA test in Saudi Arabia is less than 20$ while the cost of lab tests is about 100$, especially if not covered by insurance. Our study’s main limitation was the observational design; hence inference cannot be made—the small sample size was due to difficulty in obtaining parental consent for blood withdrawals, and the inclusion of females only. Furthermore, the use of BIA can be affected by the hydration state of the body, the intake of food or drinks before measurements, physical activity, and medical status [28]. However, we ensured that measures were standardized and all participants attended the clinic after an overnight fast of a minimum of 8 h.

## 5. Conclusions

In conclusion, a simple, non-invasive, and low-cost measure such as WC was the best tool to diagnose MetS in Saudi children and adolescents, with the highest sensitivity and a cut-off point of 81.5 cm. BMI and FM follow WC with similar sensitivity. This suggests that the combination of more than one tool is favorable for the early detection of MetS in children and young adolescents. The use of FMI by BIA is promising, but further studies should be carried out to test the predictability of MetS among a prospective cohort.

## Figures and Tables

**Table 1:** Anthropometric measurements, BIA, and biochemical data stratified by study group (normal weight, None-MetS, MetS).

Variables	Normal Weight (*n* = 108)	None-MetS (*n* = 26)	MetS (*n* = 24)
Wt	48.60 ± 8.92	68.95 ± 11.70 *p* < 0.01 *	76.31 ± 15.17 *p* < 0.01 * p < 0.01 **
Ht	154.2 ± 7.63	157.46 ± 9.13 *p* = 0.17 *	157.87 ± 8.26 *p* = 0.13 * p = 0.86 **
BMI	20.28 ± 2.76	27.74 ± 3.54 *p* < 0.01 *	30.40 ± 4.37 *p* < 0.01 * p < 0.05 **
WC	65.2 ± 6.68	84.53 ± 9.86 *p* < 0.01 *	91.58 ± 9.52 *p* < 0.01 * p < 0.01 **
BFP-SFT	28.84 ± 2.72	30.26 ± 1.74 *p* < 0.05 *	29.84 ± 1.73 *p* = 0.10 * p = 0.48 **
BFP	27.11 ± 4.94	37.31 ± 4.90 *p* < 0.01 *	39.70 ± 6.30 *p* < 0.01 * p = 0.122 **
FM	13.47 ± 4.49	25.68 ± 6.15 *p* < 0.01 *	30.50 ± 8.72 *p* < 0.01 * p < 0.05 **
FFM	35.16 ± 5.29	42.70 ± 6.22 *p* < 0.01 *	45.51 ± 8.70 *p* < 0.01 * p = 0.152 **
TBW	25.74 ± 3.87	31.27 ± 4.55 *p* < 0.01 *	33.31 ± 6.37 *p* < 0.01 * p = 0.155 **
FBG	91.44 ± 6.75	94.15 ± 12.20 *p* = 0.362 *	103.04 ± 11.80 *p* < 0.01 * p < 0.05 **
HDL	46.48 ± 7.51	44.26 ± 12.59 *p* = 0.389 *	34.79 ± 5.35 *p* < 0.01 * p < 0.01 **
TG	67.80 ± 16.13	84.76 ± 20.33 *p* = 0.161 *	123.66 ± 70.70 *p* < 0.01 * p < 0.01 **
SBP	118 ± 10.00	123.26 ± 12.76 *p* = 0.116 *	133.50 ± 12.45 *p* < 0.01 * p < 0.01 **
DBP	69.64 ± 8.99	70.92 ± 9.26 *p* = 0.649 *	78.91 ± 11.70 *p* < 0.01 * p < 0.01 **

Wt: weight; Ht: height; BMI: Body mass index; WC: waist circumference; BFP-SFT: Body fat percentage using skinfold thickness; BFP: Body fat percentage using Bioelectrical impedance; FM: fat mass; FFM: fat-free mass; TBW: total body water; FBG: fasting blood glucose; HDL: High-Density Lipoprotein; TG: Triglycerides; SBP: Systolic Blood Pressure; DBP: Diastolic Blood Pressure; Mets: Metabolic syndrome cases; * Significance versus controls, ** Significance versus None-MetS.

**Table 2:** Correlation of anthropometric measurements and BIA components with the components of MetS in the None-MetS and MetS groups.

MetS Components		Anthropometric Measurements						BIA Components			
		BMI	HC	WHR	MAC	MAMA	BFP-SFT	BFP	FMI	FFM	TBW
None-MetS group											
WC	r	0.47 *	0.65 **	0.58 **	0.47 *	0.34	−0.24	0.09	0.22	0.49 *	0.57 **
FBG	r	0.11	0.36	0.05	0.27	0.37	−0.09	0.12	−0.03	−0.29	0.11
HDL	r	−0.25	0.07	−0.14	−0.29	−0.25	0.18	0.11	−0.11	−0.44 *	−0.03
TG	r	0.09	0.14	0.15	0.11	0.18	−0.27	−0.00	0.14	0.36	0.25
SBP	r	−0.13	−0.04	−0.34	0.05	−0.10	0.22	−0.06	−0.02	0.11	0.07
DBP	r	−0.08	−0.05	0.12	0.31	0.18	−0.11	0.07	−0.06	−0.20	0.05
MetS group											
WC	r	0.54 **	0.67 **	0.45 *	0.05	−0.08	−0.57 **	0.14	0.35	0.50 *	0.38
FBG	r	0.07	0.26	0.28	−0.03	−0.20	−0.43 *	0.01	0.15	0.32	0.22
HDL	r	0.15	−0.09	0.21	0.09	0.11	−0.18	0.11	0.15	0.08	−0.10
TG	r	0.23	0.32	0.01	−0.19	−0.29	−0.32	0.04	0.16	0.24	0.31
SBP	r	0.64 **	−0.09	−0.46 *	0.20	0.22	0.50 *	0.32	0.16	−0.37	−0.27
DBP	r	0.48 *	0.01	−0.10	0.06	0.16	0.38	0.35	0.29	−0.14	−0.12

* Correlation is significant at the level of 0.05 (2-tailed). ** Correlation is significant at the level of 0.01 (2-tailed). BMI: Body mass index; HC: Hip circumference; WHR: waist-hip ratio; MAC: Mid-arm circumference; MAMA: mid-arm muscle area; BFP-SFT: fat percentage using skinfold thickness; FMI: fat mass index; FFM: fat-free mass; TBW: total body water; WC: waist circumference; FBG: fasting blood glucose; HDL: high-density lipoprotein; TG: triglycerides; SBP: systolic blood pressure; DBP: diastolic blood pressure.

**Table 3:** Sensitivity and Specificity of BMI, WC and anthropometric measures, BIA tests, and their cut-off points for diagnosing Metabolic Syndrome.

Measures	Sensitivity	Specificity	AUC (95% CI)	Cut-Off Point
BMI (kg/m^2^)	79%	80.4%	0.84 (0.75–0.93)	26.9
WC (cm)	87.5%	72.5%	0.86 (0.77–0.94)	81.5
HC (cm)	75%	72.5%	0.81 (0.72–0.91)	100.0
BFP-SFT(%)	62.5%	43.1%	0.49 (0.35–0.62)	29.9
MAMA (cm^2^)	75%	60.8%	0.74 (0.62–0.86)	26.0
BFP	79.2%	60.8%	0.79 (0.68–0.89)	34.7
FMI	83.3%	60.8%	0.82 (0.73–0.92)	8.8
FFMI	79.2%	72.5%	0.80 (0.69–0.91)	16.6
TBW	70.8%	56.9%	0.72 (0.59–0.85)	29.3

BMI: Body mass index; WC: waist circumference; HC: Hip circumference; BFP-SFT: fat percentage using skinfold thickness; MAMA: mid-arm muscle area; FMI: fat mass index; FFMI: fat-free mass index; TBW: total body water.

## Data Availability

The raw data supporting the conclusions of this article will be made available by the authors, without undue reservation, to any qualified researcher.
